# Elemental Status and Lipid Peroxidation in the Blood of Children with Endemic Fluorosis

**DOI:** 10.1007/s12011-020-02243-3

**Published:** 2020-06-17

**Authors:** Halyna Tkachenko, Natalia Kurhaluk, Natalia Skaletska, Viktor Maksin, Zbigniew Osadowski

**Affiliations:** 1 Department of Biology, Institute of Biology and Earth Sciences,, Pomeranian University in Słupsk, Arciszewski Str. 22b, 76-200 Słupsk, Poland; 2 Danylo Halytskyy Lviv National Medical University, Lviv, Ukraine; 3 National University of Life and Environmental Sciences of Ukraine, Kyiv, Ukraine

**Keywords:** Fluorosis, Oxidative stress, Lipid peroxidation, Chemical elements, Blood, Ukraine

## Abstract

The study aimed to assess the levels of trace elements, minerals, and toxic elements as well as lipid peroxidation biomarkers (lipid acyl hydroperoxides, 2-thiobarbituric acid reactive substances (TBARS)) in the blood of children with chronic fluorosis from endemic fluorosis areas (Sosnivka village, Lviv region, western Ukraine). The results were compared with healthy children from Staryi Sambir (Lviv region, western Ukraine), whose drinking water contained permissible levels (< 1 ppm) of fluoride. Thirty-one children from the Sosnivka village in the Lviv region, including 16 females and 15 males aged 7–10 years, with clinically diagnosed fluorosis, were recruited for the study. The children had been exposed to fluoride (> 1.5 ppm) through drinking water for more than 5 years. In the blood, eight macro- and microelements (calcium, zinc, potassium, iron, copper, selenium, manganese, chromium), five additional elements (sulfur, bromine, chlorine, nickel, strontium), and four toxic elements (lead, mercury, cadmium, mercury) were assessed with the X-ray fluorescence method. The results of our study demonstrated a 14-fold decrease in the copper level, a 2.5-fold decrease in the calcium and zinc levels, and a 2-fold decrease in the selenium level in the blood of children with chronic fluorosis compared with the healthy children from the non-fluorosis area. In turn, a 1.7- and 1.4-fold increase in the strontium and lead content, respectively, was noted. The sulfur, chlorine, potassium, calcium, copper, zinc, and selenium levels in the blood samples of children with chronic fluorosis were lower than the reference value. The children had higher blood TBARS levels, while the acyl hydroperoxide levels were non-significantly increased in comparison with healthy children living in the non-fluorosis area. Additionally, the bromine level was correlated positively with the selenium level and acyl hydroperoxides. However, more studies are needed to clarify the relationship between blood mineral status, oxidative stress biomarkers, and chronic fluorosis.

## Introduction

Although fluoride is considered an essential trace element, given its role in imparting stability to teeth and bones, chronic exposure to (> 1 ppm) fluoride is known to cause toxic effects [8, 33, 38, 55]. Long-term exposure to high levels of fluoride is a serious health problem in many parts of the world where drinking water contains more than 1–1.5 ppm of fluoride [33, 38, 48, 61].

Fluorosis is a well-defined clinical entity characterized by toxic effects of high fluoride intake on teeth, bones, and soft tissues [9, 24, 34]. In addition to its well-known effects on the skeleton and teeth, fluorosis can also adversely affect many tissues and organs, such as the liver and kidneys, with a broad array of symptoms and various pathological changes [27, 41]. Fluoride can cross cell membranes by simple diffusion and enter soft tissues [61]. The liver is one of the target organs attacked by fluoride. Numerous studies have revealed that excessive amounts of fluoride disturb the metabolic processes and detoxication capabilities of the liver [16]. Fluoride-induced necrosis, modifications of membrane lipids, and apoptosis in hepatocytes are associated with oxidative stress [19]. Kidneys play a prominent role in fluoride metabolism, as 50–80% of fluoride is removed via urinary excretion [23]. There was a close correlation between fluoride intake and renal injury. Fluoride-intoxicated rats showed an increased generation of reactive oxygen species (ROS) and lipid peroxidation in the kidneys [22]. In endemic fluorosis areas, drinking water with fluoride levels over 2.0 ppm can cause damage to the liver and kidney functions in children [56].

Recent studies have shown that fluoride exerts different cellular effects in time-, concentration-, and cell type–dependent manner on the cell machinery leading to cell death, apoptosis, and/or necrosis both in vivo and in vitro [2]. The main toxic effect of fluoride in cells consists of its interaction with enzymes [1]. Fluoride at micromolar levels is considered an effective anabolic agent promoting cell proliferation, whereas millimolar concentrations inhibit several enzymes, including phosphatases, both in vivo and in vitro [2, 30]. Fluoride can interact with a wide range of cellular processes such as gene expression, cell cycle, proliferation and migration, respiration, metabolism, ion transport, secretion, endocytosis, apoptosis/necrosis, and oxidative stress, and that these mechanisms are involved in a wide variety of signaling pathways [2].

Oxygen radical generation and lipid peroxidation have even been proposed to be an important mediating factor in the detrimental effects of chronic fluoride toxicity [17, 35]. However, how the whole body effects are produced is still unclear, and efforts to prevent and treat fluorosis by therapeutic measures have had only limited success [17].

Children living in territories with increased fluoride very often exhibit problems with normal physical maturity and bone formation as a result of exposure at the sensitive developmental stages, particularly the pre- and postnatal ontogenesis period, the first year of life, and during puberty period [12, 54]. Clinical symptoms in children include rachitis, osteoporosis, and disorders of the calcium homeostasis balance [12, 49].

In Central Europe, groundwater resources that exceed the upper guideline value of 1.5 ppm are widespread, and dental fluorosis associated with high fluoride concentrations in water has been reported in Ukraine, Moldova, and Hungary [12, 14, 15, 36, 64]. The information about the prevalence of fluorosis in Ukraine demonstrates that the relationships between fluorosis and fluoride concentrations in water are not simple [12]. Although it is often the case that waters containing > 1.5 ppm cause disease, the disease also occurs in areas where water fluoride contents are below 1.5 ppm and this may be due to other water chemistry factors, other non-water sources of fluoride, and dietary or physiological factors in the areas concerned [12].

In Ukraine, an assessment of two industrial regions, Chervonohrad in the west and Kharkiv-Dnipro-Donetsk-Zaporizhia in the central-eastern part of Ukraine conducted by Fordyce and Vrana (2001) and Fordyce and co-workers (2007) revealed that sources related to coal mining resulted in enhanced fluoride in the environment of Chervonohrad, but had little impact on water fluoride concentrations in Kharkiv-Dnipro-Donetsk-Zaporizhia. These findings were incorporated into the national risk assessment for Ukraine [11, 12]. In the Chervonohrad Mining District (Lviv region, Ukraine), high-fluoride waters associated with tectonically active fault zones and mining contamination result in dental fluorosis in the local population (64% prevalence rate). Alternative lower fluoride waters have been supplied to the public in recent years, but the disease is still endemic in the region. Defluoridation technologies may be helpful in this area. We performed the current study in this endemic fluorosis area in the Lviv region (western part of Ukraine).

Minerals and trace elements are necessary for both physiological and biochemical functions. Many disorders in the organism are related to altered serum mineral and trace element levels. Deficiency of essential trace elements or minerals and excess of potentially harmful trace elements or minerals are both known to have adverse effects in the general population [10]. Therefore, this study aimed to assess the levels of trace elements, minerals, and toxic elements as well as lipid peroxidation biomarkers in the blood of children with chronic fluorosis from endemic fluorosis areas (Sosnivka village, Lviv region, western Ukraine, 50° 17′ 40″ N 24° 15′ 00″ E). The results were compared with healthy children from the Staryi Sambir (Lviv region, western Ukraine, 49° 26′ 28″ N 23° 00′ 29″ E), whose drinking water contained permissible levels (< 1 ppm) of fluoride.

## Materials and Methods

### Subjects

Thirty-one children from the Sosnivka village (Lviv region), including 16 females (mean height 1.33 ± 0.02 m and mean bodyweight 30.3 ± 1.67 kg) and 15 males (1.31 ± 0.02 m and 28.88 ± 1.75 kg) aged 7–10 years, with clinically defined fluorosis were recruited for the study, with written consent from their parents. These children had been exposed to fluoride (> 1.5 ppm) through drinking water for more than 5 years. Physical examination was performed in all children. The children were also examined for having mottled tooth enamel, which is one of the diagnostic criteria of endemic fluorosis (according to Dean’s index). Body weight and height were recorded as well. The exclusion criteria of the study were the presence of any known cardiac and lung diseases, the use of cardiac drugs, diabetes mellitus, chronic renal disorders, and hepatic diseases. The participants who had taken any vitamin or mineral supplements for at least 2 weeks before blood samples were also excluded. Informed written consent was obtained from all parents. The study protocol was approved by the Institutional Human Ethical Committee (Danylo Halytskyy Lviv National Medical University, Lviv, Ukraine, No. 5 of May 26, 2014). Fifteen healthy children, i.e., 5 females (mean height 1.40 ± 0.02 m and mean bodyweight 33.12 ± 2.66 kg) and 9 males (1.38 ± 0.02 m and 32.37 ± 2.14 kg) in the age range of 7–11 years (8.09 ± 0.33 m and 7.95 ± 0.29 kg) from the Staryi Sambir city of Lviv region (western Ukraine), whose drinking water contained permissible levels (< 1 ppm) of fluoride, served as controls. The clinical history of the children was recorded.

### Blood Sample Collection

After overnight fasting, blood samples of the subjects were collected by venipuncture into K_3_-EDTA and 3.8% sodium citrate tubes. Plasma and buffy coat were removed by centrifugation at 3000 rpm for 15 min. Erythrocytes were washed 3 times with buffered saline, and the packed cells were then aliquoted for further analysis.

### Biochemical Measurements

#### Lipid Acyl Hydroperoxide Assay

The acyl hydroperoxide level was assessed in the plasma sample with the method proposed by Kamyshnikov (2004). To 0.2 mL of plasma, 4 mL of a “heptane-isopropanol” mixture was added and vortexed vigorously. Then, 1 mL of HCl (pH 2.0) and 2 mL of heptane reagent were added, vortexed, and centrifuged at 3000 rpm for 5 min. The lipid hydroperoxide level was read spectrophotometrically at 233 nm and expressed as E_233_ per milliliter. A mixture of distilled water was used in the blank samples [20].

#### 2-Thiobarbituric Acid Reactive Substance Assay

The level of lipid peroxidation was determined by quantifying the concentration of 2-thiobarbituric acid reacting substances (TBARS) with the Kamyshnikov (2004) method for determination of the malonic dialdehyde (MDA) concentration. This method is based on the reaction of the degradation of the lipid peroxidation product, MDA, with 2-thiobarbituric acid (TBA) at high temperature and acidity to generate a colored adduct that is measured spectrophotometrically. The μmol of MDA per liter was calculated using 1.56·10^5^ mM^−1^ cm^−1^ as the extinction coefficient [20].

#### X-ray Fluorescence Analysis of Blood

The levels of eight macro- and microelements (calcium, zinc, potassium, iron, copper, selenium, manganese, chromium), five additional elements (sulfur, bromine, chlorine, nickel, strontium), and four toxic elements (lead, mercury, cadmium, mercury) were assessed in the blood of each child with the use of the X-ray fluorescence assay according to *X-ray fluorescence analysis* (2000) [57]. The total number of analyzed indices was 576. The assessment of the levels of chemical elements in the blood plasma was conducted closely with experts of the Scientific and Technical Center “VIRIA Ltd.” (Kyiv, Ukraine). Plasma elemental analyses were conducted using an X-ray fluorescence spectrometer ElvaX (ElvaX, Kyiv, Ukraine). Calibrations were performed before the first use of the spectrometer for sample analysis each day.

#### Statistical Analysis

The results are expressed as mean ± S.E.M. All variables were tested for normal distribution using the Kolmogorov-Smirnov test (*p* > 0.05). The significance of differences between levels of oxidative stress biomarkers and concentrations of chemical elements (significance level, *p* < 0.05) was examined using the Kruskal-Wallis test by ranks. Correlations between the parameters at the set significance level were evaluated using Spearman’s correlation analysis [60]. All statistical calculations were performed on separate data from each individual with STATISTICA 8.0 software (StatSoft, Krakow, Poland).

## Results

The anthropometric data of the controls and fluorosis-affected children are shown in Table 1. There were no significant differences between the children with fluorosis and the controls in terms of the age, height, body weight, chest circumferences, mid-upper arm muscle circumferences, hip circumferences, and head circumferences.

**Table 1:** Anthropometric data of the controls and fluorosis-affected children (*M* ± *m*)

Anthropometric parameters	Non-fluorosis children	Chronic fluorosis children
Age (years)	8.02 ± 0.31	8.17 ± 0.34
Sex (M/F)	9/5	15/16
Height, m	1.39 ± 0.03	1.32 ± 0.02
Bodyweight, kg	32.72 ± 2.39	29.55 ± 1.72
Chest circumference, cm	65.81 ± 2.24	62.87 ± 1.60
Mid-upper arm muscle circumference, cm	20.83 ± 0.89	20.85 ± 1.14
Hip circumference maximum, cm	37.81 ± 1.59	37.05 ± 1.53
Head circumference, cm	27.86 ± 0.97	27.23 ± 0.99

**Fig. 1:**
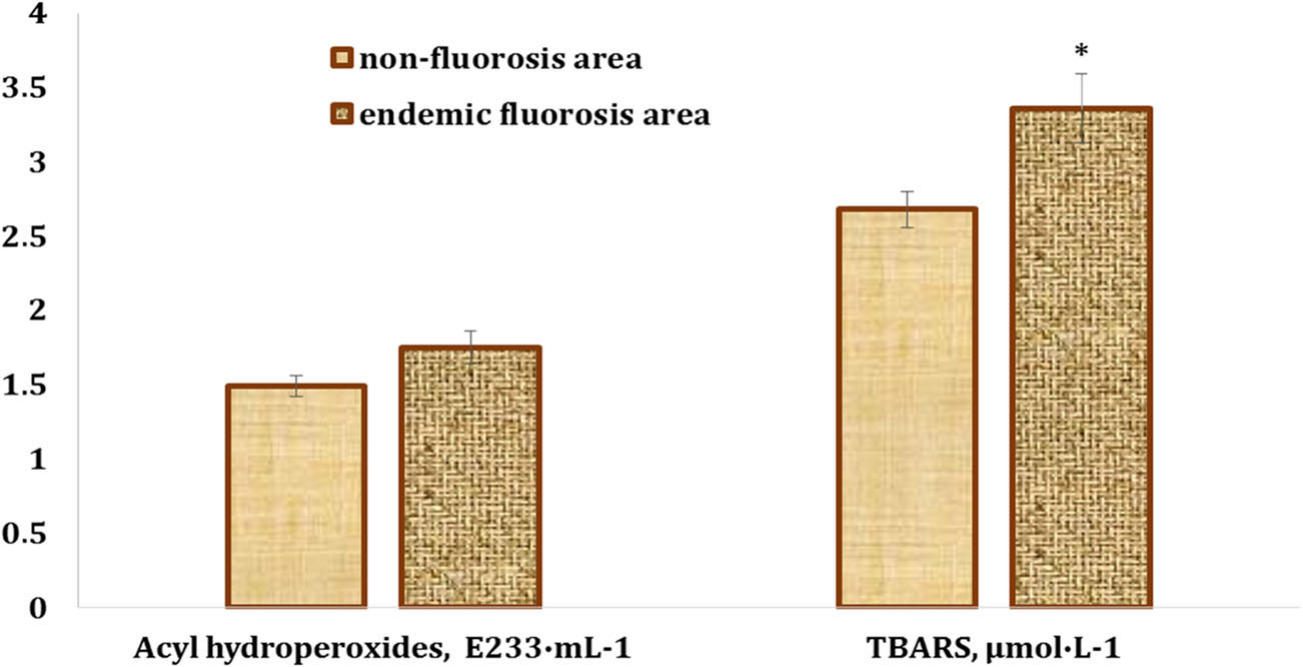
Levels of acyl hydroperoxides and TBARS in the blood of children living in endemic fluorosis areas (Sosnivka village, Sokal district, Lviv region) and healthy children from the non-fluorosis areas (Staryi Sambir city, Staryi Sambir district, Lviv region). The asterisk indicates that changes are statistically significant compared with the group of children from the non-fluorosis areas (*p* < 0.05)

There was a non-significant 17.5% increase (*p* > 0.05) in the primary products of lipid peroxidation (acyl hydroperoxides) in the blood of children from the endemic fluorosis areas (Sosnivka village), compared with the values obtained in the blood of the healthy children from the non-fluorosis area (Fig. 1). Moreover, the children with chronic fluorosis had by 25% higher blood TBARS levels (*p* < 0.05) than the healthy subjects living in the non-fluorosis areas (Staryi Sambir city) (Fig. 1).

In the current study, the levels of eight macro- and microelements (calcium, zinc, potassium, iron, copper, selenium, manganese, chromium), five additional elements (sulfur, bromine, chlorine, nickel, strontium), and four toxic elements (lead, mercury, cadmium, mercury) were assessed and presented in Table 1. The serum S levels (641.78 ± 81.62 μg/mL vs. 1035.19 ± 51.83 μg/mL, respectively, decreased by 38%, *p* < 0.01), Cl levels (1458.306 ± 169.466 μg/mL vs. 2561.93 ± 253.83 μg/mL, respectively, decreased by 43%, *p* < 0.05), K levels (117.53 ± 14.58 μg/mL vs. 141.84 ± 8.94 μg/mL, respectively, decreased by 17%, *p* < 0.05), Ca levels (36.94 ± 4.43 μg/mL vs. 91.64 ± 3.06 μg/mL, respectively, decreased by 60%, *p* < 0.01), Cu levels (0.248 ± 0.038 μg/mL vs. 3.488 ± 0.458 μg/mL, respectively, decreased by 93%, *p* < 0.01), Zn levels (0.665 ± 0.072 μg/mL vs. 0.665 ± 0.072 μg/mL, respectively, decreased by 58%, *p* < 0.01), and Se levels (0.041 ± 0.005 mg/mL vs. 0.074 ± 0.006 mg/mL, respectively, decreased by 45%, *p* < 0.01) were significantly lower in the children with chronic fluorosis than in the controls (Table 2). There were no statistically significant differences in the serum levels of Fe, Ni, Br, Cr, and Mn between the fluorosis group and the control group. Moreover, the serum Sr level was statistically significantly higher (0.0781 ± 0.0168 μg/mL vs. 0.046 ± 0.0057 μg/mL, respectively, decreased by 70%, *p* < 0.05) in the children with chronic fluorosis than in the controls, whereas the Hg, Cd, and Pb levels were statistically non-significant (Table 2).

**Table 2:** Comparison of the serum levels of trace elements, minerals, and toxic heavy metals in non-fluorosis children and chronic fluorosis children, μg/mL (*M* ± *m*)

Chemical elements	Non-fluorosis children	Chronic fluorosis children	Reference values (according to http://www.viria.com.ua)
	*М* ± *m*	*M* ± *m*	
Sulfur, S	1035.19 ± 51.83	641.78 ± 81.62**	1050–1200
Chlorine, Cl	2561.93 ± 253.83	1458.306 ± 169.466*	3400–3800
Potassium, K	141.84 ± 8.94	117.53 ± 14.58*	140–207
Calcium, Ca	91.64 ± 3.06	36.94 ± 4.43**	90–110
Iron, Fe	1.479 ± 0.133	1.96 ± 0.35	0.6–1.6
Nickel, Ni	0.024 ± 0.0023	0.0237 ± 0.0027	0.02–0.03
Bromine, Br	237.22 ± 53.75	248.068 ± 64.992	50–1500
Copper, Cu	3.488 ± 0.458	0.248 ± 0.038**	1.3–16
Zinc, Zn	0.665 ± 0.072	0.277 ± 0.0756**	0.7–1.2
Chrome, Cr	0.0653 ± 0.024	0.0475 ± 0.0064	0.03–0.12
Manganese, Mn	0.0607 ± 0.0071	0.053 ± 0.0066	0.04–0.16
Selenium, Se	0.074 ± 0.006	0.041 ± 0.005**	0.07–0.15
Strontium, Sr	0.046 ± 0.0057	0.0781 ± 0.0168*	0.04–0.13
Mercury, Hg	0.014 ± 0.0027	0.0119 ± 0.00187	0.01–0.05
Cadmium, Cd	0.01 ± 0.0011	0.01 ± 0.0011	0.01–0.027
Lead, Pb	0.0446 ± 0.0073	0.064 ± 0.015	0.05–0.2

*р < 0.05; **р < 0.001 compared with the value of children from the non-fluorosis area

The serum S, Cl, K, Ca, Cu, Zn, and Se levels in the children with chronic fluorosis were lower but the Fe levels were higher than the reference values. In the control group, the S, Cl, and Zn levels were lower than the reference values (Table 2).

The correlation analysis is presented in Table 3. In the blood samples of the children from the non-fluorosis areas, the sulfur level was correlated positively with the TBARS level (*r* = 0.581, *p* = 0.029) and inversely with both Br (*r* = − 0.638, *p* = 0.010) and Hg (*r* = − 0.584, *p* = 0.022). The potassium level was correlated positively with the lead level (*r* = 0.556, *p* = 0.039) and inversely with the chromium level (*r* = − 0.659, *p* = 0.039). The nickel level was correlated inversely with copper (*r* = − 0.612, *p* = 0.015) and manganese (*r* = − 0.565, *p* = 0.028), while the manganese level correlated positively with the chromium level (*r* = 0.593, *p* = 0.020) (Table 3).

**Table 3:** Correlation analysis between the chemical elements and oxidative stress biomarker levels in the blood of chronic fluorosis children and healthy children from the non-fluorosis areas

Relations	Spearman’s correlation coefficients, *r*	t(N-2)	*p* level
Non-fluorosis children			
S vs. Br	− 0.638	− 2.992	0.010
S vs. Hg	− 0.584	− 2.592	0.022
S vs. TBARS	0.581	2.472	0.029
K vs. Cr	− 0.659	− 3.160	0.008
K vs. Pb	0.556	2.316	0.039
Ni vs. Cu	− 0.612	− 2.788	0.015
Ni vs. Mn	− 0.565	− 2.467	0.028
Cr vs. Mn	0.533	2.268	0.041
Sr vs. Hg	0.593	2.652	0.020
Chronic fluorosis–affected children			
S vs. Cu	0.761	5.862	0.000
S vs. Zn	0.732	5.378	0.000
S vs. Se	0.478	2.718	0.012
Cl vs. K	0.382	2.065	0.049
K vs. Zn	− 0.564	− 3.413	0.002
Cu vs. Zn	0.701	4.909	0.000
Zn vs. Br	0.459	2.586	0.016
Zn vs. Se	0.626	4.015	0.000
Br vs. Se	0.394	2.144	0.042
Br vs. AHP	0.415	2.279	0.031

In the blood samples of the children with chronic fluorosis, the sulfur level was correlated positively with copper (*r* = 0.761, *p* = 0.000), zinc (*r* = 0.7321, *p* = 0.000), and selenium (*r* = 0.478, *p* = 0.012). The potassium level was correlated positively with chlorine (*r* = 0.382, *p* = 0.049) and inversely with the zinc level (*r* = − 0.564, *p* = 0.002). The zinc level was correlated positively with copper (*r* = 0.701, *p* = 0.000), bromine (*r* = 0.459, *p* = 0.016), and selenium levels (*r* = 0.626, *p* = 0.000), while the bromine level correlated positively with the content of selenium (*r* = 0.394, *p* = 0.042) and acyl hydroperoxides (*r* = 0.415, *p* = 0.031) (Table 3). Additionally, the height of the children was correlated positively with calcium (*r* = 0.200, *p* = 0.021), zinc (*r* = 0.253, *p* = 0.004), and strontium levels (*r* = 0.223, *p* = 0.011), whereas their age was correlated positively with the content of zinc (*r* = 0.253, p = 0.004) and selenium (*r* = 0.173, *p* = 0.048).

## Discussion

The current study has focused on lipid peroxidation biomarkers in the blood of children with chronic fluorosis living in endemic fluorosis areas. The results of the present study showed that children with chronic fluorosis had higher blood TBARS levels, while the acyl hydroperoxide levels were non-significantly increased, in comparison with the healthy children living in the non-fluorosis areas (Fig. 1). Moreover, the results also demonstrated a 14-fold decrease in the copper level, a 2.5-fold decrease in the calcium and zinc levels, and a 2-fold decrease in the selenium level in the blood of children with chronic fluorosis, compared with the healthy children from the non-fluorosis areas. In turn, there was a 1.7- and 1.4-fold increase in the strontium and lead content, respectively. The sulfur, chlorine, potassium, calcium, copper, zinc, and selenium levels in the blood samples of children with chronic fluorosis were lower than the reference value (Table 2). Only two correlative relationships were observed between element levels and lipid peroxidation biomarkers. In the blood samples of the children with chronic fluorosis, the blood acyl hydroperoxide levels were correlated with the bromine levels (*r* = 0.415, *p* = 0.031) (Table 3). TBARS level was positively correlated with sulfur level (*r* = 0.581, *p* = 0.029) in the blood of healthy children from the non-fluorosis areas. Moreover, there were no significant differences in the anthropometric data between the fluorosis-affected children and the controls (Table 1).

It is known that fluoride is an inducer of oxidative stress and modulates intracellular redox homeostasis, lipid peroxidation, and protein oxidation. It also alters gene expression and causes apoptosis [2]. The association between fluoride toxicity and elevated oxidative stress has been widely reported in humans and experimental animals [2, 43, 44, 51, 53, 58, 59, 63]. Fluoride exposure increases the generation of anion superoxide (O_2_^−∙^) [13]. An increased O_2_^−∙^ concentration and its downstream consequences such as hydrogen peroxide, peroxynitrite, and hydroxyl radicals seem particularly important in mediating the effects of fluoride. Moreover, fluoride increases NO generation and can react with superoxide to form peroxynitrite and with thiols and metal centers in proteins to form nitrosyl adducts [26]. It has also been shown to interfere with the disulfide-bond formation and result in the accumulation of misfolded proteins in the endoplasmic reticulum, causing stress and ROS production [2]. It is known that excessive ROS production leads to macromolecule oxidation, resulting in a free radical attack on membrane phospholipids with resulting membrane damage via induction of lipid peroxidation, mitochondrial membrane depolarization, and apoptosis [2]. The results of our study revealed an increase in the TBARS level, i.e., a biomarker of lipid peroxidation, in the blood of the children with chronic fluorosis (Fig. 1).

In our study, decreased serum levels of Cu and Zn in children with chronic fluorosis were observed (Table 2). In our opinion, the low Cu and Zn levels in the fluorosis-affected children may be associated with fluoride-induced oxidative stress. Ersoy and co-workers (2011) also showed decreased serum levels of Zn in patients with chronic fluorosis. Increased utilization of Cu and Zn for counteracting fluoride-induced oxidative stress may also be, at least partially, responsible for the decrease in the blood level of these elements [10]. Singh (1984) also showed significantly reduced liver and kidney levels of Zn, Cu, and Mn in fluoride-treated mice. Zn is transported in an albumin-bound form [45]. Bennis and co-workers (1993) showed that plasma proteins decreased in chronic fluoride poisoning [4]. Therefore, decreased plasma proteins might be a factor for the decreased serum Zn concentration in fluorosis patients. Additionally, reduced gastrointestinal absorption and tissue-specific absorption of Zn may also have contributory effects [10].

Oxidative stress is a recognized mode of action of fluoride exposure that has been observed in vitro in several types of cells and in vivo in soft tissues such as the liver, kidney, brain, lungs, and testes in animals and people living in areas of endemic fluorosis [2]. Fluoride is thought to inhibit the activity of antioxidant enzymes such as superoxide dismutase (SOD), glutathione peroxidase, and catalase [13]. Moreover, fluoride can alter glutathione levels, often resulting in excessive production of ROS at the mitochondrial level, leading to damage to cellular components [2, 32, 37].

Antioxidant treatment consistently protects cells from lipid peroxidation caused by fluoride exposure, suggesting that oxidative/nitrosative damage is the major mode of action of fluoride [18, 32]. In our previous study [47, 50], we revealed that the oxidative stress in the blood of fluorosis-affected children caused impairments in the antioxidant defenses. Specifically, superoxide dismutase, catalase, glutathione reductase, and glutathione peroxidase activity were decreased. Decreased blood Cu and Zn levels may play the main role in the decrease in SOD activity. Uauy and co-workers (1985) demonstrated decreased Cu/Zn-SOD activity in 17 infants during Cu deprivation, which may be improved when Cu is added to the diet. SOD was correlated with plasma Cu; thus, the erythrocyte SOD activity is a good marker of Cu nutrition in humans [52].

We also found reduced serum selenium levels in children with fluorosis (Table 2). Selenium is a cofactor required for the activity of several seleno-enzymes involved in stress-induced responses and maintenance of high tissue antioxidant levels [3]. Selenium in a certain concentration range was involved in excretion of high fluoride, regulation of free radicals and lipid metabolism disorder, and promotion of recovery in rats with fluorosis. Moreover, Se was able to antagonize high fluoride levels as well as delay and reduce the occurrence of skeletal fluorosis in rats [62]. The antioxidative nature of selenium coupled with its reversal effect on metabolic enzymes in the brain of mice treated with fluoride suggests its use as an antidote agent against fluorosis [39]. The ameliorative effect of selenium is related to its role in various physiological functions, including its role as a biologically active antioxidant. Selenium is an essential component of antioxidant enzyme glutathione peroxidase (GSH-Px). In a certain concentration range, it was involved in excreting high fluorine levels, adjusting free radicals and lipid metabolism disorders, and elevating the antioxidant capacity of fluorosis. Results reported by Reddy and co-workers (2009) indicated that selenium could antagonize long-term fluoride exposure. The optimum level of selenium for the antagonistic effect is 1.5 mg/L. Thus, selenium plays a critical role in the maintenance of the proper blood antioxidant capacity [39]. In our previous study, low selenium levels induced a decrease in blood GSH-Px activity in the serum of children with chronic fluorosis [47, 50].

Although there are many studies on the effect of fluoride on trace elements in experimental animals, few studies on serum trace element levels have been carried out in patients with endemic fluorosis [10]. In humans, there are some investigations of the effects of chronic fluorosis on micronutrient mineral levels. Meral and co-workers (2004) found a significant decrease in the serum levels of Cu, Zn, Mn, and Mg in 15 fluorosis patients [31]. Similarly, Chen and co-workers (2002) found a significant decrease in the serum concentrations of Ca, Mg, and Cu, and an increase in serum Fe in skeletal fluorosis patients [5]. Ersoy and co-workers (2011) determined the serum levels of trace elements, including serum Cu and Zn as well as serum levels of minerals such as Ca, P, Mg, Na, and K in patients with endemic fluorosis. The study group consisted of 30 patients with endemic fluorosis. Thirty age-, gender-, and body mass index–matched healthy volunteers constituted the control group. The serum Cu, Zn, and Mg levels were significantly lower in chronic fluorosis patients than in the controls. There were no statistically significant differences in the serum levels of Na, K, Ca, and P between the fluorosis patients and the control group [10]. Ersoy and co-workers (2011) found that chronic fluorosis was associated with reduced serum levels of Cu, Zn, and Mg. Singh and Kanwar (1981) investigated changes in Cu and Fe in certain tissues in experimental fluorosis. These researchers reported reduced concentrations of Cu in the liver, kidney, and bone of fluoride-intoxicated mice along with increased Fe in these organs [46]. Some research conducted in numerous animal models and humans has shown that Cu deficiency can cause an increase in the plasma cholesterol concentration [7, 21].

Increased serum levels of strontium and lead in children with chronic fluorosis were observed (Table 2). The lead and strontium levels in the drinking water in Sosnivka village were in the range of 0.001–0.023 mg/L and 0.011–2.08 mg/L, respectively. According to state sanitary norms and rules (Ukrainian state standards 2.2.4-171-10) “*Hygienic requirements for drinking water intended for human consumption*”, the maximum allowable concentration (MAC) of lead and strontium in drinking water is less than 0.01 and 7 mg/L, respectively. In our study, the lead levels in the drinking water in Sosnivka village were 2.3-fold higher than the state standards. A possible relationship between high fluoride levels in water and elevated blood lead and strontium concentrations in the children with fluorosis was suggested. Ecological associations have been reported between the use of silicofluoride compounds (sodium silicofluoride and hydrofluosilicic acid) and NaF as water fluoridation methods and elevated blood lead concentrations in children [29]. Similar results were observed in children who live in old houses supplied by fluoridated water [28]. It is, therefore, possible that the increased water lead levels observed when fluoride concentrations are between 1 and 2 mg/L could be severely aggravated in places with high fluoride concentrations in the drinking water [2]. However, a recent study found that fluoride increased blood lead concentrations and lead levels in calcified tissues of animals exposed to low levels of lead, suggesting biological interactions as a possible cause of the epidemiological relationship between high Pb levels and the fluoridation of drinking water [40]. Sr is considered to accumulate in bone and teeth because it has similar properties to the congener Ca. Sr is easily incorporated into apatite, and Sr^2+^ occupies the Ca^2+^ position in hydroxyapatite, Ca_10_(PO_4_)_6_(OH)_2_ [25, 42]. Sr has been essential for laboratory animals but not for humans. However, when incorporated into the bone, it behaves like an essential element, as it acts in the same way as Ca. Regarding the association between Sr and caries, the Sr level in enamel increased as the Sr concentration in drinking water increased, and the prevalence of caries reflected in decayed, missing, and filled surfaces was inversely related to the Sr levels in drinking water and enamel [6, 42].

## Conclusions

In conclusion, the results of our study demonstrated a 14-fold decrease in the copper level, a 2.5-fold decrease in the calcium and zinc levels, and a 2-fold decrease of the selenium level in the blood of children with chronic fluorosis, compared with the healthy children from the non-fluorosis areas. In turn, a 1.7- and 1.4-fold increase in the strontium and lead content, respectively, was noted. The sulfur, chlorine, potassium, calcium, copper, zinc, and selenium levels in the blood samples of children with chronic fluorosis were lower than the reference value. The children with chronic fluorosis had higher blood TBARS levels, while the acyl hydroperoxide levels were non-significantly increased, in comparison with the values in the healthy children living in the non-fluorosis areas. Additionally, the bromine level was correlated positively with the selenium level and acyl hydroperoxides. However, further studies are needed to clarify the relationship between blood mineral status, oxidative stress biomarkers, and chronic fluorosis.
